# Fatigue, economic security, and job satisfaction: a cross-sectional study conducted in Ningbo, China during the post-restriction period

**DOI:** 10.3389/fpubh.2026.1861160

**Published:** 2026-07-15

**Authors:** Jingjing Ma, Qihang Xu, Lu Shi, Yiqing Zhang

**Affiliations:** 1 Department of Nursing, Ningbo Medical Center LiHuili Hospital, Ningbo, China; 2 Department of Pharmacy, Ningbo Medical Center LiHuili Hospital, Ningbo, China; 3 Department of Rehabilitation Medicine, Ningbo Medical Center LiHuili Hospital, Ningbo, China

**Keywords:** COVID-19, economic security, fatigue, health, job satisfaction

## Abstract

**Background:**

The COVID-19 pandemic has contributed to widespread fatigue among workers, posing a significant challenge to occupational health. Grounded in Conservation of Resources (COR) theory, this study examines the association between pandemic-related fatigue and job satisfaction and investigates whether economic security serves as a moderating factor.

**Methods:**

A cross-sectional survey was conducted among employed residents in Ningbo, China, shortly after the lifting of COVID-19 restrictions. Data were collected using a COVID-19 Occupational Health Impact Questionnaire, which included demographic characteristics, COVID-19 history, employment status, the Fatigue Assessment Scale (FAS), a multidimensional Economic Security Index, and a job satisfaction measure. Multivariable logistic regression was used to assess associations and statistical interactions (moderation effects).

**Results:**

Among 1,938 participants included in the final analysis (retention rate: 98.5% of completed questionnaires), fatigue was significantly associated with lower job satisfaction (adjusted odds ratios (OR) = 0.27, 95% confidence intervals (CI): 0.22–0.34, *p* < 0.001). Although economic security was positively associated with job satisfaction (OR = 2.92, 95% CI: 2.39–3.58, *p* < 0.001), it did not significantly moderate the association between fatigue and job satisfaction (P for interaction = 0.458). The association between fatigue and lower job satisfaction was consistent across both economically secure (OR = 0.33, 95% CI: 0.24–0.44) and economically insecure (OR = 0.25, 95% CI: 0.10–0.65) subgroups.

**Conclusion:**

Fatigue is strongly associated with job dissatisfaction. In this cross-sectional study, we did not find evidence that economic security moderated this association. These findings suggest that fatigue is strongly associated with job dissatisfaction across economic strata. Future research should examine whether workplace fatigue mitigation strategies are associated with in protecting employee well-being in the post-pandemic era.

## Introduction

The COVID-19 pandemic has triggered a global public health crisis, with over 670 million confirmed cases and 6.8 million deaths globally by early 2023 (1). Healthcare systems were overwhelmed, disrupting 40% of essential health services (2), and the economic downturn has had profound societal impacts (3, 4). These cascading effects intensified psychological distress, culminating in what the WHO termed “pandemic fatigue”: diminished compliance with preventive measures, blunted risk perception, and emotional exhaustion (5).

The occupational toll of this fatigue has become a critical concern (6, 7). Among COVID-19 survivors, an estimated 35–60% report persistent symptoms, including fatigue and cognitive impairment, four months post-infection (8, 9). During 12–18 months of follow-up, 13.7–62.7% experienced deterioration in work status, including sick leave, job loss, or reduced hours (10, 11). Frontline workers, particularly healthcare staff, have been disproportionately affected (12, 13), raising concerns about post-COVID conditions contributing to workforce shortages (14).

Job satisfaction during the pandemic tells a divided story. Among teleworkers, largely college-educated and working from home, 69% reported high satisfaction, likely due to greater autonomy, flexibility, and reduced infection risk (15–17). Medical staff faced a different reality: overwhelming workloads and inadequate resources left many dissatisfied (18, 19). These patterns suggest a potential cycle: workplace stress is associated with lower job satisfaction (20).

China maintained strict containment policies from August 2021, effectively suppressing viral transmission (21). The relaxation of restrictions in January 2023 created twin occupational health crises: acute absenteeism surges and a heightened risk of chronic fatigue syndrome (22, 23). Ningbo, a major industrial hub in the Yangtze River Delta, has a large manufacturing workforce. During the Omicron wave, Ningbo experienced a high infection burden, with widespread absenteeism disrupting workplace operations. The region’s work culture is characterized by long hours and intense productivity demands, and its economy faced significant post-lockdown pressures including reduced export demand. These local conditions make Ningbo an ideal setting to examine the association between pandemic-related fatigue and job satisfaction.

Hobfoll’s Conservation of Resources (COR) theory (24) suggests that individuals strive to protect their valued resources (e.g., energy, material goods). Fatigue represents a state of resource depletion, which may increase vulnerability to further losses—including reduced job satisfaction. Conversely, economic security, as a material resource, may help individuals cope with fatigue by enabling access to recovery opportunities. The Job Demands-Resources (JD-R) model (25) complements this view by distinguishing job demands (e.g., workload) that deplete energy from job resources (e.g., economic security) that foster engagement. Together, these frameworks suggest that fatigue (a demand) and economic security (a resource) may interact to shape job satisfaction.

Despite evidence linking organizational support and psychological resources, such as resilience and self-efficacy, to work-related stress (26, 27), the moderating role of economic security in the fatigue-job satisfaction relationship remains largely untested. Most existing studies have targeted healthcare workers or specific occupational groups, overlooking the general workforce during the post-restriction period (28). Psychological resources have received far more attention than material ones (29). Moreover, whether COR theory’s resource-buffering logic holds under systemic crises is unclear (30); it is possible that even substantial economic security may not fully offset resource depletion under such conditions (31).

This study addresses these gaps in three ways. First, we examine the fatigue–job satisfaction association in a general working population (rather than a single occupational group) immediately after the lifting of COVID-19 restrictions in Ningbo. Second, we test whether economic security moderates this association. Third, we assess the applicability of COR theory’s resource-buffering hypothesis in the unique context of a systemic public health crisis. We hypothesized that: (H1) fatigue would be negatively associated with job satisfaction; (H2) economic security would be positively associated with job satisfaction; and (H3) economic security would moderate the association between fatigue and job satisfaction, such that the negative association would be weaker among those with higher economic security. The findings may offer practical guidance for organizations seeking to support employee well-being and maintain workforce stability in the post-pandemic era.

## Materials and methods

### Participants

This cross-sectional study used a community-based online survey conducted via WeChat, China’s largest social media platform, to recruit employed residents in Ningbo from January 27 to February 5, 2023. To enhance sample diversity and reach a broader cross-section of the local workforce, the survey invitation was distributed across multiple diverse WeChat groups targeting different population segments, including community resident groups, industry-specific professional groups (e.g., healthcare, education, manufacturing), and local interest-based communities. The 10-day data collection period was a deliberate design choice. During this time, Ningbo was experiencing one of its largest epidemic waves, with infection rates increasing rapidly following the lifting of COVID-19 restrictions (17). This brief yet focused window allowed us to capture respondents’ experiences precisely during the peak Omicron surge, minimizing temporal heterogeneity that would have arisen from a longer collection period. By concentrating data collection into a short timeframe, we minimized temporal confounding. We ensured that all participants were surveyed under comparable external conditions (e.g., similar policy environments, infection risks, and workplace disruptions).

### Eligibility criteria

Participants were eligible for the study if they met the following criteria:

#### Inclusion Criteria

(1) Residency: employed residents of Ningbo.
(2) Age: aged 18 years or older.
(3) Language and Literacy: able to read and write in Chinese to complete the study scales and questionnaires.
(4) Informed Consent: willing to provide informed consent by completing the online consent form before starting the survey.

#### Exclusion criteria

(1) Psychiatric Disorders: diagnosed with major psychiatric conditions (e.g., severe depression, bipolar disorder, schizophrenia, or anxiety disorders).
(2) Chronic Diseases: suffering from chronic illnesses that may manifest as fatigue (e.g., chronic fatigue syndrome, fibromyalgia, cancer, autoimmune diseases, or severe cardiovascular diseases).
(3) Inability to Participate: unable to complete the requirements due to cognitive, linguistic, or physical limitations.

### Recruitment and data collection

Participants were recruited online via WeChat, China’s most popular social network, which has more than 1.29 billion active users. The survey was distributed via a quick response code, which was shared in targeted WeChat groups and public accounts known to have a high concentration of Ningbo residents. To reach a diverse audience, a multi-channel recruitment strategy was employed:

(1) Social Media: the survey was promoted on platforms such as Weibo and QQ, with posts targeting adult users in Ningbo.
(2) Email Lists: We collaborated with local organizations and universities to distribute the survey to their members.
(3) Professional Forums: the survey was shared on industry-specific forums and community groups to include participants from various professional backgrounds.

The survey briefly described its purpose, background, and procedures. It also explained the voluntary nature of participation, confidentiality, anonymity, and precautions for completing the questionnaire. The informed consent form was attached to the first page of the questionnaire. Participants were required to review the consent information and indicate their agreement by selecting a designated option before proceeding; those who did not agree or did not meet the eligibility criteria were instructed to withdraw. Participants were informed that individuals with psychiatric disorders or chronic diseases that might manifest as fatigue were not eligible for the study. They were instructed to withdraw from the questionnaire if they had such conditions.

### Survey procedure

Respondents were asked to complete a 15–20-min online survey covering personal characteristics, fatigue, job satisfaction, and COVID-19-related experiences. To enhance the reliability of self-reported data, we implemented the following measures:

(1) Ensured participant anonymity to encourage honest responses;
(2) Designed clear and concise questions to improve understanding;
(3) Conducted pilot testing to gather feedback and refine the questionnaire;
(4) Implemented human verification and attention checks throughout the study to ensure data quality.

### Sample size calculation

The target population was adults aged ≥18 years in Ningbo, China. The initial sample size target (*N* = 1,800) was calculated based on the Ningbo resident population to achieve a 3% margin of error at the 95% confidence level. For regression analysis, the “events per variable” (EPV) rule recommends 10–15 events per predictor. With 14 predictors, at least 150–225 participants with job dissatisfaction were required. Our final sample included 881 such participants (45.5%), yielding an EPV of 58.7—well above the recommended minimum. *Post hoc* power analysis using G*Power 3.1 indicated that, with our final sample of 1,938 participants and 14 variables in the fully adjusted model, we had >80% power to detect a small interaction effect (f^2^ = 0.02) for the fatigue × economic security term at *α* = 0.05.

### Measures

The survey instrument was entitled “COVID-19 Occupational Health Impact Survey.” It consisted of four parts. A structured questionnaire was developed for this study, combining adapted scales with investigator-designed items.

(1) This study collected multidimensional survey data encompassing demographic characteristics (including age, gender, marital status, highest educational attainment, height, weight, body mass index, smoking, and alcohol consumption), COVID-19 infection history (infection status and symptom duration), as well as current occupational status (covering occupation type, working years, weekly working hours, monthly income). Symptom duration was assessed among participants who reported COVID-19 infection. Participants were asked: “How long did your COVID-19 symptoms last?” Response options were: (1) 1–2 days, (2) 3–4 days, (3) ≥ 5 days, or (4) not applicable (asymptomatic or no infection).
(2) Fatigue was measured using the 10-item FAS (32), a validated instrument assessing physical and psychological fatigue. A cut-off of ≥22 defined problematic fatigue, consistent with prior COVID-19 studies (33). In this study, the FAS demonstrated high internal consistency (Cronbach’s *α* = 0.869).
(3) Economic security was measured using a multidimensional composite index adapted from the Economic Security Index (ESI) framework developed by Hacker and colleagues (34). Participants were asked to provide a self-assessment based on four thresholds derived from the literature (34, 35): liquidity resilience (able to cover ≥3 months of basic expenses), debt sustainability (debt-to-income ratio ≤40%), income stability (income decline <25%), and healthcare preparedness (able to afford out-of-pocket medical expenses). Participants who rated themselves as secure on at least three of the four dimensions were classified as economically secure. Consistent with the dual-cutoff counting method (36) and formative measurement models (37), this “3 of 4” threshold reflects a robust level of economic security. Traditional psychometric validation (e.g., factor analysis) is conceptually inappropriate for this index (38).
(4) Job satisfaction was assessed using a single-item instrument (39). Participants rated their current employment experience on an anchored 5-point Likert scale, ranging from 1 (strongly dissatisfied) to 5 (strongly satisfied). Single-item job satisfaction scales have been shown to correlate strongly (r = 0.60–0.72) with multi-item measures, with good criterion validity and test–retest reliability (40–42). This concise approach prioritizes real-time data capture while minimizing respondent burden, consistent with survey best practices in public health emergency settings.

To ensure data quality, two attention check questions were embedded in the survey: (1) ‘Please select “Agree” for this question to confirm you are paying attention’; and (2) ‘For this item, choose “Strongly disagree” as your response.’ Respondents who failed either attention check were excluded from analysis. Additionally, responses completed in less than five minutes (indicating speeding) or exhibiting straight-lining patterns (selecting the same response option for all items in a scale) were flagged as invalid and removed.

### Conceptual framework and outcome definitions

This study examined the relationships among three types of variables. The independent variable was fatigue, assessed using the Fatigue Assessment Scale (FAS) and classified as present (FAS total score ≥22) or absent. The dependent variable was job satisfaction, measured by a single-item 5-point Likert scale and classified as satisfied (responses of “satisfied” or “strongly satisfied”) or dissatisfied (“strongly dissatisfied,” “dissatisfied” or neutral). The moderating variable was economic security, classified as secure or insecure based on the multidimensional index described above. We hypothesized that economic security moderates the association between fatigue and job satisfaction, such that the negative association between fatigue and job satisfaction would be weaker among economically secure individuals (Supplementary Table S1).

### Statistical analysis

Continuous variables were presented as mean ± standard deviation, and categorical variables as frequencies and percentages. Group comparisons were conducted using independent t-tests for continuous variables and chi-square tests for categorical variables.

Binary logistic regression examined associations between job satisfaction and two key factors: fatigue and economic security. Three sequential models were constructed: Model 1 (unadjusted), Model 2 (adjusted for demographic and lifestyle factors: gender, age, marital status, education, smoking, drinking, BMI), and Model 3 (fully adjusted, additionally including COVID-19 infection, symptom duration, occupation, income, and working life). Job satisfaction was coded as 1 = satisfied, 0 = dissatisfied.

Effect modification by economic security was assessed through stratified analyses and interaction testing. Stratified logistic regression models estimated the association between fatigue and job satisfaction within economic security subgroups (secure vs. insecure). A multiplicative interaction term (fatigue × economic security) was included in Model 3 and evaluated using likelihood ratio tests (P for interaction < 0.05, indicating a significant interaction).

Sensitivity analyses tested the robustness of findings to alternative classifications of the neutral category in job satisfaction: (1) liberal classification (satisfied: 3–5; dissatisfied: 1–2), and (2) strict classification (neutral excluded; satisfied: 4–5; dissatisfied: 1–2) (Supplementary Table S1).

All tests were two-sided with significance set at *p* < 0.05. Results are reported as ORs with 95% CIs. For fatigue, ORs < 1 indicate a negative association with job satisfaction; for economic security, ORs > 1 indicate a positive association. Multicollinearity was assessed using variance inflation factors (VIFs). All VIFs ranged from 1.013 to 1.811, well below the conventional threshold of 5, indicating no problematic multicollinearity.

Bivariate associations among key binary variables were assessed using Pearson correlation coefficients. Analyses were performed using R (version 4.4.0) and Zstats (version 1.0).

### Ethics approval and consent to participate

This study was conducted in accordance with the Declaration of Helsinki. All methods were carried out in accordance with relevant guidelines and regulations. This study was approved by the Ethics Committee of Ningbo Medical Center LiHuili Hospital (approval no: KYSB2022SL246). All procedures followed relevant guidelines and regulations. All participants provided informed consent before the survey; they were assured that their personal information would be kept confidential.

## Results

### Descriptive statistics

A total of 1,968 questionnaires were collected. After data cleaning, 30 respondents were excluded for the following reasons: 12 failed attention checks; 10 completed the survey in less than 5 min (speeding); 5 exhibited straight-lining patterns (selecting the same response option for all items on a scale); and 3 had missing data on key variables. The final analytic sample comprised 1,938 respondents. The distribution of job satisfaction across the five response categories was as follows: strongly dissatisfied (*n* = 317, 16.4%), dissatisfied (*n* = 481, 24.8%), neutral (*n* = 83, 4.3%), satisfied (*n* = 865, 44.6%), and strongly satisfied (*n* = 192, 9.9%). Key demographic and clinical characteristics are presented in Table 1. Females constituted the majority of the study population (56.3%). The 18–35 age group was the most represented (58.0%). Most participants were married (71.72%) and had attained middle-school or lower levels of education (46.75%). Health-related metrics revealed that 68.11% maintained normal BMI ranges (18.5–24.9 kg/m^2^). Notably, 75.03% reported prior COVID-19 infection, and 24.72% were medical professionals. Workplace characteristics displayed marked economic stratification: 41.12% earned less than 6,000 CNY monthly (< 884 USD) (Table 1).

**Table 1:** Demographic characteristics of participants stratified by job satisfaction status (*n* = 1,938).

Variables	Total (*n* = 1,938)	Job satisfied (*n* = 1,057)	Job dissatisfied (*n* = 881)	Statistic	*p*
Gender, *n* (%)				χ^2^ = 19.46	<0.001
Male	847 (43.70)	414 (48.88)	433 (51.12)		
Female	1,091 (56.30)	643 (58.94)	448 (41.06)		
Age, *n* (%)				χ^2^ = 16.07	<0.001
18–35	1,124 (58.00)	586 (52.14)	538 (47.86)		
36–65	665 (34.31)	402 (60.45)	263 (39.55)		
>65	149 (7.69)	69 (46.31)	80 (53.69)		
BMI (kg/m^2^), mean ± SD	23.05 ± 3.06	22.90 ± 3.18	23.23 ± 2.90	t = −2.40	0.016
Marital status, *n* (%)				χ^2^ = 0.09	0.77
Married	1,390 (71.72)	761 (54.75)	629 (45.25)		
Unmarried	548 (28.28)	296 (54.01)	252 (45.99)		
Education, *n* (%)				χ^2^ = 86.25	<0.001
Middle school or below	906 (46.75)	411 (45.36)	495 (54.64)		
High school and college	744 (38.39)	504 (67.74)	240 (32.26)		
Bachelor’s degree	288 (14.86)	142 (49.31)	146 (50.69)		
Smoking, *n* (%)				χ^2^ = 71.27	<0.001
Yes	731 (37.72)	309 (42.27)	422 (57.73)		
No	1,207 (62.28)	748 (61.97)	459 (38.03)		
Drinking, *n* (%)				χ^2^ = 37.37	<0.001
Yes	722 (37.25)	329 (45.57)	393 (54.43)		
No	1,216 (62.75)	728 (59.87)	488 (40.13)		
Infection COVID-19, *n* (%)				χ^2^ = 3.20	0.074
Yes	1,454 (75.03)	810 (55.71)	644 (44.29)		
No	484 (24.97)	247 (51.03)	237 (48.97)		
Symptom duration, *n* (%)				χ^2^ = 6.64	0.084
1–2 days	878 (45.30)	451 (51.37)	427 (48.63)		
3–4 days	525 (27.09)	302 (57.52)	223 (42.48)		
≥ 5 days	293 (15.12)	165 (56.31)	128 (43.69)		
Not applicable	242 (12.49)	139 (57.44)	103 (42.56)		
Occupation, *n* (%)				χ^2^ = 150.02	<0.001
Teacher	123 (6.35)	60 (48.78)	63 (51.22)		
Medical worker	479 (24.72)	374 (78.08)	105 (21.92)		
Self-employed household	291 (15.02)	117 (40.21)	174 (59.79)		
Worker	563 (29.05)	263 (46.71)	300 (53.29)		
Other	482 (24.87)	243 (50.41)	239 (49.59)		
Income (RMB/USD), *n* (%)				χ^2^ = 21.14	<0.001
< 6,000 CNY (< 884 USD)	797 (41.12)	401 (50.31)	396 (49.69)		
6,000–8,000 CNY (884–1,178 USD)	566 (29.21)	299 (52.83)	267 (47.17)		
8,001–10,000 CNY (1178–1,473 USD)	345 (17.80)	207 (60.00)	138 (40.00)		
>10,000 CNY (> 1,473 USD)	230 (11.87)	150 (65.22)	80 (34.78)		
Working life, *n* (%)				χ^2^ = 7.66	0.053
< 5 years	500 (25.80)	264 (52.80)	236 (47.20)		
5–10 years	399 (20.59)	231 (57.89)	168 (42.11)		
11–15 years	531 (27.40)	269 (50.66)	262 (49.34)		
>15 years	508 (26.21)	293 (57.68)	215 (42.32)		

t: t-test, Z: Mann–Whitney test, χ^2^: Chi-square test. SD: standard deviation, M: Median, Q₁: 1st Quartile, Q₃: 3rd Quartile. Values are *n* (%). Percentages in the “Job satisfied” and “Job dissatisfied” columns represent row proportions (i.e., % within each gender/age/education group). USD equivalents are based on the 2023 average exchange rate (1 USD ≈ 6.79 CNY; values rounded).

### Main study variables by job satisfaction status

Table 2 presents the distribution of the main study variables (economic security and fatigue) by job satisfaction status. Among economically secure participants, 66.5% were job satisfied compared to 36.7% among economically insecure participants (*p* < 0.001). The median fatigue score was 29.00 in both satisfied and dissatisfied groups (*p* = 0.024) (Table 2).

**Table 2:** Distribution of main study variables by job satisfaction status (*n* = 1938).

Variables	Total (*n* = 1938)	Job satisfied (*n* = 1,057)	Job dissatisfied (*n* = 881)	Statistic	*p*
Economic security, *n* (%)				χ^2^ = 166.89	<0.001
Secure	1,161 (59.91)	772 (66.49)	389 (33.51)		
Insecure	777 (40.09)	285 (36.68)	492 (63.32)		
Fatigue, M (Q₁, Q₃)	29.00 (21.00, 32.00)	29.00 (21.00, 32.00)	29.00 (20.00, 32.00)	Z = −2.26	0.024

χ^2^ test for economic security; Mann–Whitney Z test for fatigue.

### The association between fatigue and job satisfaction

Table 3 demonstrates a consistent negative association between fatigue and job satisfaction across regression models. In the fully adjusted model (Model 3), fatigue was significantly associated with lower job satisfaction (OR = 0.27, 95% CI: 0.22–0.34, *p* < 0.001). The full results of the multivariable logistic regression model, including coefficients for all covariates, are provided in Supplementary Table S2.

**Table 3:** Association between job satisfaction and fatigue.

Variables	Model 1		Model 2		Model 3	
	OR (95%CI)	*p*	OR (95%CI)	*p*	OR (95%CI)	*p*
Fatigue						
No	1.00 (Reference)		1.00 (Reference)		1.00 (Reference)	
Yes	0.40 (0.32–0.49)	<0.001	0.31 (0.25–0.39)	<0.001	0.27 (0.22–0.34)	<0.001

OR: Odds Ratio, CI: Confidence Interval. Model 1: Crude. Model 2: Adjust: Gender, Age, Marital status, Education, Smoking, Drinking, BMI. Model 3: Adjust: Gender, Age, Marital status, Education, Smoking, Drinking, Infection COVID-19, Duration of symptoms, Occupation, Income RMB, Working life, BMI. Job satisfaction was dichotomized as satisfied (1) vs. dissatisfied (0), with ‘satisfied’ as the outcome event. Fatigue was coded as fatigued (1) vs. non-fatigued (0), with ‘non-fatigued’ as the reference category. Odds ratios (ORs) < 1 indicate a negative association between fatigue and job satisfaction. For complete model results including all covariates, see Supplementary Table S2.

### The association between economic security and job satisfaction

Table 4 shows a significant positive association between economic security and job satisfaction across all models. In the fully adjusted model (Model 3), economically secure workers had higher odds of job satisfaction compared to economically insecure workers (OR = 2.92, 95% CI: 2.39–3.58, *p* < 0.001). The full results are provided in Supplementary Table S3.

**Table 4:** Multivariable logistic regression analysis of economic security as a predictor of job satisfaction (*n* = 1,938).

Variables	Model 1		Model 2		Model 3	
	OR (95%CI)	*p*	OR (95%CI)	*p*	OR (95%CI)	*p*
Economic Security						
Insecure	1.00 (Reference)		1.00 (Reference)		1.00 (Reference)	
Secure	3.43					
(2.84–4.15)	<0.001	3.07 (2.53–3.74)	<0.001	2.92		
(2.39–3.58)	<0.001					

OR: Odds Ratio, CI: Confidence Interval. Model 1: Crude. Model 2: Adjust: Gender, Age, Marital status, Education, Smoking, Drinking, BMI. Model3: Adjust: Gender, Age, Marital status, Education, Smoking, Drinking, Infection COVID-19, Duration of symptoms, Occupation, Income RMB, Working life, BMI, Economic security was defined as meeting at least 3 of 4 security criteria (see Methods for details). ORs > 1 indicate that economic security is associated with higher odds of job satisfaction. For complete model results including all covariates, see Supplementary Table S3.

### Subgroup analysis and interaction test

**Figure 1:**
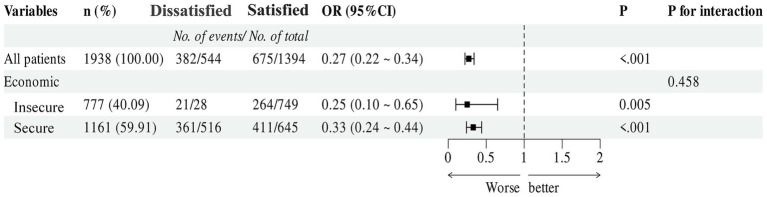
Fatigue and job satisfaction: stratified analysis by economic security. Forest plot showing the association between fatigue and job satisfaction stratified by economic security status. ORs < 1 indicate lower odds of job satisfaction among fatigued participants.

**Figure 2:**
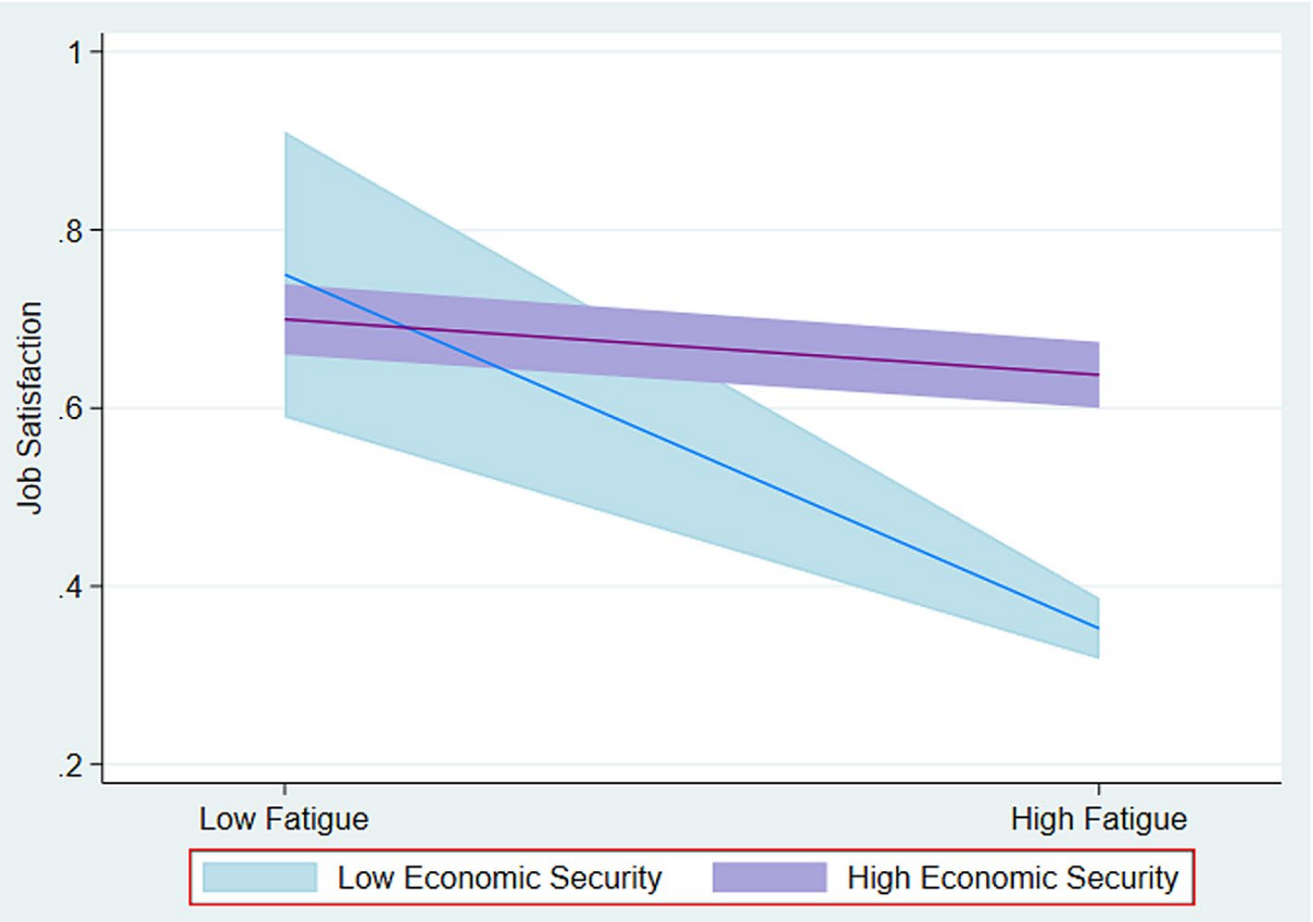
Moderation effect of economic security on the fatigue–job satisfaction relationship. The figure displays the proportion of job satisfaction among non-fatigued and fatigued participants, stratified by economic security status. The x-axis represents fatigue level as a dichotomous categorical variable (Low vs. High), so categorical labels are used. The y-axis indicates the proportion of job satisfaction (ranging from 0 to 1) within each fatigue group.

Correlation analysis revealed significant associations among fatigue, job satisfaction, and economic security (Table 5). In subgroup analyses stratified by economic security, fatigue was significantly associated with lower job satisfaction in both groups. Among economically secure participants (*n* = 1,161), fatigue was associated with lower job satisfaction (OR = 0.33, 95% CI: 0.24–0.44, *p* < 0.001). Among economically insecure participants (*n* = 777), fatigue was also associated with lower job satisfaction (OR = 0.25, 95% CI: 0.10–0.65, *p* = 0.005). The interaction term was not statistically significant (P for interaction = 0.458), indicating that the association between fatigue and job satisfaction did not differ significantly by economic security status (Figure 1). The proportion of job satisfaction decreased from 75.0% in the non-fatigued group to 35.2% in the fatigued group; among economically secure participants, the proportion decreased from 70.0 to 63.7% (Figure 2).

**Table 5:** Correlations among fatigue, job satisfaction, and economic security.

Variables	Fatigue	Job satisfaction	Economic security
Fatigue	1		
Job satisfaction	−0.197**	1	
Economic security	−0.445**	0.293**	1

Coefficients are Pearson correlation coefficients. For binary variables coded as 0/1, Pearson correlations are equivalent to phi (φ) coefficients. Variables were coded as: Fatigue (1 = fatigued, 0 = non-fatigued); Job satisfaction (1 = satisfied, 0 = dissatisfied); Economic security (1 = secure, 0 = insecure). *p < 0.05; **p < 0.01.

## Discussion

The COVID-19 pandemic has significantly affected job satisfaction and fatigue among workers (43), highlighting the importance of understanding the interplay between these factors and the potential role of economic security as a moderator. Previous research has suggested that during crises, heavy workloads, emotional exhaustion, and resource scarcity may be associated with depletion of both cognitive and material reserves, thereby potentially exacerbating fatigue through accelerated resource depletion (44, 45). Building on this evidence, the present study examined the association between fatigue and job satisfaction during the COVID-19 pandemic and investigated whether economic security moderates this association. However, our results indicate that economic security does not play a significant moderating role in the fatigue–job satisfaction association.

### Fatigue and job satisfaction

Our analysis shows that fatigue is robustly associated with lower job satisfaction. This finding held across all regression models. This association is consistent with the Job Demands-Resources (JD-R) model (46). This model distinguishes job demands from job resources. Chronic job demands are associated with exhaustion and impaired well-being in previous research (47, 48). Fatigue is considered a key indicator of such exhaustion. It may reflect reduced cognitive and emotional capacity, which is, in turn, associated with lower satisfaction with work. This pattern is consistent with the COR theory (24).

### Economic security and job satisfaction

Our findings indicate a significant association between economic instability and job satisfaction. This is consistent with previous research, which suggests that economic stability is associated with job-related well-being (24). Especially during crises like the COVID-19 pandemic, when economic instability occurs, workers from disadvantaged groups may experience greater losses in their sense of well-being (35).

Although income is one component of economic security, the latter is a multidimensional construct that captures financial vulnerability beyond current income level, including liquidity resilience, debt sustainability, and healthcare preparedness (34).

### Economic security as a non-moderator

Contrary to our hypothesis, economic security did not significantly moderate the fatigue-job satisfaction association (interaction *p* = 0.458). This null finding should be interpreted with caution. One possibility is that during a systemic crisis—characterized by widespread health threats and economic uncertainty—the negative association of fatigue with job satisfaction may be sufficiently strong that it cuts across economic strata. Alternatively, the null interaction may reflect the specific operationalization of economic security used in this study (a multidimensional objective index), which does not capture subjective financial stress. Therefore, the absence of a significant interaction does not rule out a potential buffering role of economic security.

From a theoretical perspective, this finding invites caution rather than a strong revision of existing frameworks. COR theory suggests that material resources may buffer resource loss (24). However, a public health crisis creates a unique context of widespread uncertainty, health threats, and workplace disruption. In this study, fatigue was consistently associated with lower job satisfaction regardless of economic security status. Even among economically secure workers, fatigue remained strongly associated with lower job satisfaction (OR = 0.33). This pattern suggests that the association of fatigue with job satisfaction may be pervasive across economic strata during a systemic crisis.

Unlike some pre-pandemic studies that reported a significant moderating role of economic factors (49), our study did not find evidence of such moderation. We suggest the association may be context-dependent. Systemic crises may alter the relative importance of these factors (43, 47). Further research with larger sample sizes is needed to examine whether economic security moderates the relationship between job satisfaction and fatigue under different conditions.

### Cultural and occupational context

In China, long working hours and high work intensity are common in many industries, particularly in manufacturing and technology sectors. Overtime work is often expected or implicitly encouraged as a sign of commitment. In this context, workers may be reluctant to report fatigue or seek accommodations, yet our study still found a significant association between fatigue and job dissatisfaction, highlighting the robustness of this relationship.

The lifting of COVID-19 restrictions in Ningbo (January 2023) coincided with a nationwide Omicron wave, creating unique occupational health challenges (43). This was characterized by acute absenteeism driven by widespread infections, compounded by the intense workplace pressure to resume full operations rapidly (17). This unprecedented context likely amplified both fatigue levels and its negative occupational consequences.

Our data revealed significant occupational variation in both fatigue and job satisfaction. As shown in Supplementary Table 4, medical workers had the highest proportion of high fatigue (61.2%), followed by teachers (53.7%) and other occupations (53.7%), while manufacturing workers had the lowest proportion (46.4%). This pattern is consistent with previous research documenting elevated burnout and fatigue among healthcare workers during the COVID-19 pandemic (12, 13).

Regarding job satisfaction (Table 1), medical workers paradoxically reported the highest satisfaction rate (78.1%), while self-employed workers reported the lowest satisfaction (40.2%). This finding suggests that job satisfaction is influenced by factors beyond fatigue alone, such as job security, social support, or intrinsic motivation (50, 51). The high satisfaction among medical workers may reflect their strong professional identity and sense of purpose during the crisis.

### Practical implications

If replicated in longitudinal studies, these findings would suggest that addressing fatigue may be a workplace priority, regardless of employees’ economic security status. Organizations may consider piloting fatigue screening programs and evaluating their associations with job satisfaction. Future research should compare whether universal fatigue mitigation strategies are associated with better outcomes than income-targeted approaches during public health emergencies.

## Conclusion

This study found that fatigue was strongly associated with lower job satisfaction among workers in post-lockdown Ningbo. We did not find evidence that economic security moderated this association, though this null finding warrants caution. The consistent negative association across economic strata suggests that future longitudinal research should examine whether fatigue mitigation interventions are associated with broad-based benefits for job satisfaction, regardless of workers’ economic security status.

### Limitations

This study has several limitations. First, the cross-sectional design precludes causal inference. Longitudinal studies are needed to establish temporal ordering.

Second, job satisfaction was assessed using a single-item scale. Such single-item measures may not capture the full multidimensionality of job satisfaction. Future studies should consider multi-item scales.

Third, economic security was assessed using a composite self-rating based on four predefined thresholds (liquidity resilience, debt sustainability, income stability, healthcare preparedness), which may introduce measurement simplification. Future studies should collect dimension-specific data to allow more granular validation of the counting approach.

Fourth, this study did not fully integrate infection severity into the analyses. Symptom duration was measured using a simple categorical question (1–2 days, 3–4 days, ≥5 days, not applicable). Detailed long COVID assessment (e.g., using WHO post-COVID criteria) was not feasible during the acute Omicron wave. Future studies should incorporate validated long COVID instruments.

Fifth, although we observed significant occupational differences in fatigue (χ^2^ = 185.69, *p* < 0.001) and job satisfaction (χ^2^ = 150.02, *p* < 0.001), our study was not powered to formally test whether the fatigue–job satisfaction association varies across occupations due to limited sample sizes in certain subgroups (e.g., teachers, *n* = 123; self-employed, *n* = 291). Future large-scale studies should examine sector-specific differences using stratified analyses or interaction tests.

Sixth, the Fatigue Assessment Scale (FAS) used in this study was originally developed for chronic illness populations. It measures general physical and psychological fatigue rather than work-specific fatigue or occupational burnout. Additionally, geographic generalizability is limited, as the sample was drawn solely from Ningbo, a socioeconomically advanced coastal metropolis, restricting applicability to regions with different economic and labor market structures.

## Data Availability

The raw data supporting the conclusions of this article will be made available by the authors, without undue reservation.
