# Supplementary material for: Fatigue, economic security, and job satisfaction: a cross-sectional study conducted in Ningbo, China during the post-restriction period
Source: Front Public Health. 2026 Jul 15;14:1861160. doi: 10.3389/fpubh.2026.1861160 (PMC13416354; doi:10.3389/fpubh.2026.1861160)
Supplement: Supplementary file 1 [file Table_1.docx]

| **Supplementary Table S1. Sensitivity analyses for the associations of fatigue and economic security with job satisfaction under different dichotomizations of the outcome variable** | | | | | | | | |
| --- | --- | --- | --- | --- | --- | --- | --- | --- |
| **Analysis** | **Definition of Satisfied** | **N** | **Fatigue (Yes vs. No)** | | | **Economic Security (Secure vs. Insecure)** | | |
|  |  |  | **Model 1** | **Model 2** | **Model 3** | **Model 1** | **Model 2** | **Model 3** |
|  |  |  | **OR (95% CI)** | **OR (95% CI)** | **OR (95% CI)** | **OR (95% CI)** | **OR (95% CI)** | **OR (95% CI)** |
| **Primary analysis** | **4–5 vs. 1–3** | 1938 | 0.40 (0.32–0.49)*** | 0.31 (0.25–0.39)*** | 0.27 (0.22–0.34)*** | 3.43 (2.84–4.15)*** | 3.07 (2.53–3.74)*** | 2.92 (2.39–3.58)*** |
| **(Conservative)** | **(neutral = dissatisfied)** |  |  |  |  |  |  |  |
| **Sensitivity A** | **3–5 vs. 1–2** | 1938 | 0.48 (0.38–0.59)*** | 0.39 (0.31–0.48)*** | 0.34 (0.27–0.43)*** | 2.90 (2.40–3.51)*** | 2.58 (2.12–3.14)*** | 2.45 (2.00–3.00)*** |
| **(Liberal)** | **(neutral = satisfied)** |  |  |  |  |  |  |  |
| **Sensitivity B** | **4–5 vs. 1–2** | 1855 | 0.43 (0.35–0.54)*** | 0.34 (0.27–0.42)*** | 0.30 (0.23–0.37)*** | 3.31 (2.73–4.03)*** | 2.92 (2.39–3.57)*** | 2.79 (2.27–3.44)*** |
| **(Strict)** | **(neutral excluded)** |  |  |  |  |  |  |  |
|  |  |  |  |  |  |  |  |  |
| Notes:*** P < 0.001 for all estimates Job satisfaction was measured on a 5-point scale (1 = strongly dissatisfied to 5 = strongly satisfied) Fatigue was coded as 1 = fatigued, 0 = non-fatigued, with "non-fatigued" as reference Economic security was coded as 1 = secure, 0 = insecure, with "insecure" as reference Model 1: Unadjusted Model 2: Adjust: Gender, Age, Marital status, Education, Smoking, Drinking, BMI  Model 3: Adjust: Gender, Age, Marital status, Education, Smoking, Drinking, Infection COVID-19, Duration of symptoms, Occupation, Income RMB, Working life, BMI  For Sensitivity B, 83 respondents who selected the neutral option (3) were excluded from analysis | | | | | | | | |
