# Supplementary material for: Fatigue, economic security, and job satisfaction: a cross-sectional study conducted in Ningbo, China during the post-restriction period
Source: Front Public Health. 2026 Jul 15;14:1861160. doi: 10.3389/fpubh.2026.1861160 (PMC13416354; doi:10.3389/fpubh.2026.1861160)
Supplement: Supplementary file 2 [file Table_2.docx]

| Supplementary Table S2. Full multivariable logistic regression results for factors associated with job satisfaction (Model 3, N = 1,938） | | | | | |
| --- | --- | --- | --- | --- | --- |
| **Variables** | **β** | **SE** | **Z** | **P** | **OR (95% CI)** |
| **Fatigue (Ref: No)** |  |  |  |  |  |
| **Yes** | -1.3 | 0.12 | -10.94 | <0.001 | 0.27 (0.22–0.34) |
| **Gender (Ref: Male)** |  |  |  |  |  |
| **Female** | 0.13 | 0.11 | 1.27 | 0.203 | 1.14 (0.93–1.41) |
| **Age (Ref: 18–35)** |  |  |  |  |  |
| **35–65** | 0.61 | 0.18 | 3.45 | <0.001 | 1.84 (1.30–2.60) |
| **>65** | 0.5 | 0.24 | 2.06 | 0.039 | 1.65 (1.03–2.67) |
| **Marital status (Ref: Unmarried)** |  |  |  |  |  |
| **Married** | -0.05 | 0.17 | -0.29 | 0.771 | 0.95 (0.68–1.32) |
| **Education (Ref: Middle school or below)** |  |  |  |  |  |
| **High school and college** | 0.25 | 0.14 | 1.78 | 0.075 | 1.29 (0.98–1.70) |
| **Bachelor's degree** | -0.3 | 0.17 | -1.72 | 0.086 | 0.74 (0.53–1.04) |
| **Smoking (Ref: No)** |  |  |  |  |  |
| **Yes** | 0.45 | 0.11 | 4.1 | <0.001 | 1.56 (1.26–1.94) |
| **Drinking (Ref: No)** |  |  |  |  |  |
| **Yes** | 0.13 | 0.11 | 1.19 | 0.232 | 1.14 (0.92–1.42) |
| **BMI (kg/m², continuous)** | -0.01 | 0.02 | -0.85 | 0.396 | 0.99 (0.95–1.02) |
| **Infection COVID-19 (Ref: No)** |  |  |  |  |  |
| **Yes** | -0.1 | 0.12 | -0.84 | 0.404 | 0.91 (0.72–1.14) |
| **Duration of symptoms (Ref: 1–2 days)** |  |  |  |  |  |
| **2–4 days** | 0.07 | 0.12 | 0.59 | 0.552 | 1.08 (0.85–1.37) |
| **≥5 days** | 0.14 | 0.15 | 0.92 | 0.359 | 1.15 (0.86–1.54) |
| **Not applicable** | 0.19 | 0.16 | 1.2 | 0.232 | 1.21 (0.88–1.67) |
| **Occupation (Ref: Teacher)** |  |  |  |  |  |
| **Medical worker** | 1.12 | 0.23 | 4.78 | <0.001 | 3.06 (1.94–4.85) |
| **Self-employed household** | -0.47 | 0.25 | -1.9 | 0.057 | 0.63 (0.39–1.01) |
| **Worker** | -0.08 | 0.24 | -0.35 | 0.724 | 0.92 (0.58–1.46) |
| **Other** | 0.22 | 0.28 | 0.81 | 0.419 | 1.25 (0.73–2.16) |
| **Income RMB (Ref: <6000 CNY , < 884 USD)** |  |  |  |  |  |
| **6000–8000 CNY (884–1178 USD)** | 0.16 | 0.14 | 1.09 | 0.276 | 1.17 (0.88–1.56) |
| **8000–10000 CNY (1178–1473 USD)** | 0.28 | 0.17 | 1.7 | 0.088 | 1.33 (0.96–1.83) |
| **>10000 CNY (> 1473 USD)** | 0.51 | 0.19 | 2.61 | 0.009 | 1.66 (1.13–2.42) |
| **Working life (Ref: <5 years)** |  |  |  |  |  |
| **6–10 years** | 0.01 | 0.16 | 0.07 | 0.943 | 1.01 (0.74–1.38) |
| **11–15 years** | -0.63 | 0.21 | -3.01 | 0.003 | 0.53 (0.35–0.80) |
| **>15 years** | -0.08 | 0.23 | -0.34 | 0.737 | 0.93 (0.59–1.44) |
| Note: This table corresponds to Model 3 in Table 2 and includes all covariates adjusted in the fully adjusted model.  Outcome variable is job satisfaction (0 = dissatisfied, 1 = satisfied). β = regression coefficient; SE = standard error; OR = odds ratio; CI = confidence interval. | | | | | |
