# Supplementary material for: Fatigue, economic security, and job satisfaction: a cross-sectional study conducted in Ningbo, China during the post-restriction period
Source: Front Public Health. 2026 Jul 15;14:1861160. doi: 10.3389/fpubh.2026.1861160 (PMC13416354; doi:10.3389/fpubh.2026.1861160)
Supplement: Supplementary file 3 [file Table_3.docx]

| **Supplementary Table S3. Full multivariable logistic regression results for factors associated with job satisfaction (Model 3, N = 1,938)** | | | | | |
| --- | --- | --- | --- | --- | --- |
| **Variables** | **β** | **SE** | **Z** | **P** | **OR (95% CI)** |
| **Economic Security (Ref: Insecure)** |  |  |  |  |  |
| **Yes** | 1.07 | 0.10 | 10.36 | <.001 | 2.92 (2.39 –3.58) |
| **Gender (Ref: Male)** |  |  |  |  |  |
| **Female** | 0.10 | 0.10 | 0.93 | 0.352 | 1.10 (0.90– 1.35) |
| **Age (Ref: 18–35)** |  |  |  |  |  |
| **35–65** | 0.65 | 0.18 | 3.70 | <.001 | 1.92 (1.36– 2.72) |
| **>65** | 0.64 | 0.24 | 2.65 | 0.008 | 1.89 (1.18–3.02) |
| **Marital status (Ref: Unmarried)** |  |  |  |  |  |
| **Married** | –0.06 | 0.17 | –0.35 | 0.729 | 0.94 (0.68 – 1.31) |
| **Education (Ref: Middle school or below)** |  |  |  |  |  |
| **High school and college** | 0.23 | 0.14 | 1.64 | 0.102 | 1.26 (0.96 – 1.65) |
| **Bachelor's degree** | –0.24 | 0.17 | –1.41 | 0.160 | 0.78 (0.56 – 1.10) |
| **Smoking (Ref: No)** |  |  |  |  |  |
| **Yes** | 0.39 | 0.11 | 3.65 | <.001 | 1.48 (1.20 – 1.83) |
| **Drinking (Ref: No)** |  |  |  |  |  |
| **Yes** | 0.07 | 0.11 | 0.68 | 0.499 | 1.08 (0.87 – 1.33) |
| **BMI (kg/m², continuous)** | –0.01 | 0.02 | –0.63 | 0.526 | 0.99 (0.96 – 1.02) |
| **Infection COVID–19 (Ref: No)** |  |  |  |  |  |
| **Yes** | –0.04 | 0.12 | –0.32 | 0.749 | 0.96 (0.77 – 1.21) |
| **Duration of symptoms (Ref: 1–2 days)** |  |  |  |  |  |
| **2–4 days** | 0.08 | 0.12 | 0.63 | 0.529 | 1.08 (0.85 – 1.37) |
| **≥5 days** | 0.13 | 0.15 | 0.85 | 0.396 | 1.14 (0.85 – 1.52) |
| **Not applicable** | 0.25 | 0.16 | 1.55 | 0.121 | 1.28 (0.94 – 1.76) |
| **Occupation (Ref: Teacher)** |  |  |  |  |  |
| **Medical worker** | 0.72 | 0.23 | 3.08 | 0.002 | 2.06 (1.30 – 3.26) |
| **Self–employed household** | –0.44 | 0.24 | –1.80 | 0.072 | 0.64 (0.40 – 1.04) |
| **Worker** | –0.12 | 0.23 | –0.50 | 0.615 | 0.89 (0.56 – 1.41) |
| **Other** | 0.12 | 0.28 | 0.42 | 0.675 | 1.12 (0.65 – 1.94) |
| **Income RMB (Ref: <6000 CNY , < 884 USD)** |  |  |  |  |  |
| **6000–8000 CNY (884–1178 USD)** | 0.10 | 0.14 | 0.68 | 0.498 | 1.10 (0.83 – 1.46) |
| **8000–10000 CNY (1178–1473 USD)** | 0.18 | 0.17 | 1.07 | 0.285 | 1.19 (0.86 – 1.65) |
| **>10000 CNY (> 1473 USD)** | 0.43 | 0.19 | 2.20 | 0.028 | 1.53 (1.05 – 2.24) |
| **Working life (Ref: <5 years)** |  |  |  |  |  |
| **6–10 years** | –0.06 | 0.16 | –0.38 | 0.707 | 0.94 (0.69 – 1.29) |
| **11–15 years** | –0.69 | 0.21 | –3.28 | 0.001 | 0.50 (0.33 – 0.76) |
| **>15 years** | –0.01 | 0.23 | –0.05 | 0.962 | 0.99 (0.63 – 1.55) |
| Note: This table corresponds to Model 3 in Table 3 and includes all covariates adjusted in the fully adjusted model. Outcome variable is job satisfaction (0 = dissatisfied, 1 = satisfied). β = regression coefficient; SE = standard error; OR = odds ratio; CI = confidence interval. | | | | | |
