# Supplementary material for: Fatigue, economic security, and job satisfaction: a cross-sectional study conducted in Ningbo, China during the post-restriction period
Source: Front Public Health. 2026 Jul 15;14:1861160. doi: 10.3389/fpubh.2026.1861160 (PMC13416354; doi:10.3389/fpubh.2026.1861160)
Supplement: Supplementary file 4 [file Table_4.docx]

| **Supplementary Table S4. High fatigue (score ≥22) by occupational sector** | | |
| --- | --- | --- |
| **Occupation** | **N** | **High fatigue (≥22), n (%)** |
| **Medical worker** | 479 | 293 (61.2%) |
| **Teacher** | 123 | 66 (53.7%) |
| **Other** | 482 | 259 (53.7%) |
| **Self-employed** | 291 | 155 (53.3%) |
| **Worker** | 563 | 261 (46.4%) |
| **Total** | 1938 | 1,034 (53.4%) |
| Note: χ² = 185.69, p < 0.001. Values represent crude (unadjusted) proportions. Adjusted analyses (controlling for gender, age, BMI, marital status, education, smoking, drinking, COVID-19 infection, symptom duration, income, and working life) are presented in the main manuscript. | | |
